# Estimation and Discriminability of Doppler Ultrasound Fetal Heart Rate Variability Measures

**DOI:** 10.3389/frai.2021.674238

**Published:** 2021-08-20

**Authors:** Johann Vargas-Calixto, Philip Warrick, Robert Kearney

**Affiliations:** ^1^ Department of Biomedical Engineering, McGill University, Montreal, QC, Canada; ^2^ PeriGen Inc., Montreal, QC, Canada

**Keywords:** fetal heart rate, cardiotocography, autocorrelation, Doppler ultrasound, classification, fetal heart rate variability

## Abstract

Continuous electronic fetal monitoring and the access to databases of fetal heart rate (FHR) data have sparked the application of machine learning classifiers to identify fetal pathologies. However, most fetal heart rate data are acquired using Doppler ultrasound (DUS). DUS signals use autocorrelation (AC) to estimate the average heartbeat period within a window. In consequence, DUS FHR signals loses high frequency information to an extent that depends on the length of the AC window. We examined the effect of this on the estimation bias and discriminability of frequency domain features: low frequency power (LF: 0.03–0.15 Hz), movement frequency power (MF: 0.15–0.5 Hz), high frequency power (HF: 0.5–1 Hz), the LF/(MF + HF) ratio, and the nonlinear approximate entropy (ApEn) as a function of AC window length and signal to noise ratio. We found that the average discriminability loss across all evaluated AC window lengths and SNRs was 10.99% for LF 14.23% for MF, 13.33% for the HF, 10.39% for the LF/(MF + HF) ratio, and 24.17% for ApEn. This indicates that the frequency domain features are more robust to the AC method and additive noise than the ApEn. This is likely because additive noise increases the irregularity of the signals, which results in an overestimation of ApEn. In conclusion, our study found that the LF features are the most robust to the effects of the AC method and noise. Future studies should investigate the effect of other variables such as signal drop, gestational age, and the length of the analysis window on the estimation of fHRV features and their discriminability.

## Introduction

Continuous electronic fetal monitoring (EFM) is a standard of care during the antepartum and intrapartum periods (American College of Obstetricians and Gynecologists, 2014). EFM involves measuring two signals: fetal heart rate (FHR) and uterine pressure (UP). These two signals make up what is known as cardiotocography (CTG). Non-invasive Doppler ultrasound (DUS) is the preferred FHR acquisition method in clinical settings (Kupka et al., 2020). Uterine pressure is commonly acquired using external sensors that measure the tension in the maternal abdominal wall (Smyth, 1957). There are other acquisition methods: fetal scalp electrocardiography (ECG) for FHR; and intrauterine probes for uterine pressure (Ayres-De-Campos and Nogueira-Reis, 2016). However, these methods are invasive and are typically used only when external monitoring is not possible.

During the antepartum period, FHR monitoring has been shown to provide information about fetal reactivity (Romano et al., 2006) and abnormalities such as intrauterine growth restriction (Signorini et al., 2003; Signorini et al., 2020). During labour, the fetus is exposed to repeated periods of hypoxia during uterine contractions (McNamara and Johnson, 1995). If severe enough, sustained hypoxia can lead to metabolic acidosis and hypoxic-ischemic encephalopathy (HIE). Clinicians assess the risk of acidosis and HIE by visually monitoring the EFM for characteristic FHR patterns such as the baseline, accelerations, and decelerations (American College of Obstetricians and Gynecologists, 2014; Lear et al., 2018). Nevertheless, visual assessment of FHR tracings has low specificity and sensitivity as well as high intra- and inter-observer variability (Farquhar et al., 2020). The application of computerized analysis to quantify FHR signals has been proposed to reduce intra- and inter-observer variability (Keith and Greene, 1994). However, recent studies show that automating the analysis of classical FHR patterns does not yield a significant improvement in the detection of acidosis or HIE (Elliott et al., 2010; Clark et al., 2017; Campanile et al., 2018).

It is thought that the development of new FHR indices reflecting the physiological phenomena of acidosis and HIE could improve the ability to identify fetuses at risk (Hamilton and Warrick, 2013). In this context, fetal heart rate variability (fHRV) shows promise to be an important marker of fetal status (Signorini et al., 2003). Heart rate variability (HRV) quantifies variations in the length of the RR interval in successive heartbeats and has been widely used in adults (Acharya et al., 2006). Most HRV analysis algorithms are based on RR intervals derived from ECG signals (Ramshur 2010). However, it is difficult to use these methods for fetal monitoring since DUS measures of FHR do not provide the RR intervals. For this reason, clinical use of fHRV is generally limited to the visual analysis of FHR variations around its baseline.

The DUS transducer emits an ultrasound wave towards the fetal heart. The movement of the fetal heart changes the frequency of the reflected wave due to the Doppler effect (Hamelmann et al., 2020). As a result, both the amplitude and phase of the reflected wave are modulated and consequently its envelope varies with a frequency related to FHR (Hamelmann et al., 2020). FHR is then estimated from the autocorrelation (AC) of the DUS signal envelope computed over a window several seconds long. The AC, which measures the similarity of the signal to itself across time, will have a maximum at a lag equal to the average RR interval (Kupka et al., 2020). FHR is estimated as the inverse of this average RR interval. Fetal monitors use sliding windows to estimate FHR at a uniform sampling rate.

As a result of the averaging associated with computing the AC method, estimates of fHRV features derived from DUS ($FHR_{DUS}$) will differ from those estimated from RR intervals ($FHR_{RRI}$). Thus, estimates of power spectral density (PSD) features computed from uniformly sampled HR have been shown to overestimate the low frequency power and underestimate the high frequency power compared to those computed from non-uniformly sampled RR intervals (Clifford and Tarassenko, 2005). Thus, $FHR_{DUS}$ estimates are smoother and have less high frequency (HF) power. Attempts to reconstruct $FHR_{RRI}$ from $FHR_{DUS}$ have not been able to recover the short-term variability features associated with HF fHRV (Cesarelli et al., 2007; Kupka et al., 2020). The errors in fHRV estimates computed for $FHR_{DUS}$ will depend on the AC window length. Longer windows yield more averaging and thus underestimate HF power. Unfortunately, manufacturers of CTG monitors do not disclose the details of their AC algorithms, making it difficult to compare the estimation errors of different monitors.

More sophisticated methods have been proposed to improve the estimation of $FHR_{DUS}$ (Alnuaimi et al., 2017). Peters et al. (2004) used a low-pass filter to roughly estimate the location of the cardiac cycles and defined an AC window that contained only two heart cycles, improving the estimation of spectral features. Similarly, Jezewski et al. (2011) proposed an algorithm which varied AC window length according to an adaptive estimate of beat-to-beat intervals. Valderrama et al. (2019) developed an open-source AC method that optimizes the peak search parameters using Bayesian optimization. Another approach by Katebi et al. (2020) applied unsupervised hidden semi-Markov models to segment the DUS signal for FHR estimation. This approach was able to recover HF features that were very close to those of fECG (Katebi et al., 2020). Despite their improvements, none of these sophisticated methods have yet been applied in bedside monitors (Jezewski et al., 2017).

The availability of large cohorts of perinatal EFM recordings has motivated the development of machine learning (ML) classifiers to improve the early detection of fetal distress and reduce the risk of further injury (Georgieva et al., 2017; Petrozziello et al., 2019). Thus, fHRV features from $FHR_{DUS}$ have been used to identify fetuses with fetal abnormalities using ML and deep learning (DL) (Georgieva et al., 2017; Petrozziello et al., 2019; Signorini et al., 2020). Nevertheless, the discriminability of these algorithms will be adversely affected by errors in the estimation of fHRV features. Durosier et al. (2014) found that the root mean square of the successive differences (RMSSD) of FHR estimated from $FHR_{DUS}$ had worse discriminability than when estimated from $FHR_{RRI}$. Similarly, it has been suggested that HF $FHR_{DUS}$ features are less discriminative than from $FHR_{RRI}$ (De Jonckheere et al., 2019). The decreased discriminability of fHRV features, along with the undisclosed differences in commercial $FHR_{DUS}$ estimation algorithms, will likely affect the performance of ML classifiers.

This paper analyzes the influence of the AC window length and noise on the estimation and discriminability of some important linear and non-linear fHRV features. These features considered have all been proposed previously for the detection of fetal distress (Signorini et al., 2003). Despite the development of the new sophisticated AC algorithms, we focus on the classical AC method which is the basis of current monitors. The rationale behind this is the desire understand the properties of fHRV computed from EFM data acquired at bedside with current monitors. Thus, our objectives are twofold: 1) To determine how fHRV features computed from $FHR_{DUS}$ differ from those computed from $FHR_{RRI}$; and 2) To evaluate how these differences influence the ability to classify signals with different fHRV properties. To do so, we explored how different AC window lengths and noise levels affect the estimation of linear PSD features and the nonlinear feature approximate entropy (ApEn). Our results showed that the low frequency power (LF) is least affected by the AC and noise, while ApEn is affected the most. Furthermore, we examined how the discriminability of each feature varied with AC window length and noise and showed that LF was the most stable feature.

## Materials and Methods

This section describes the methods used for:

1) Simulating RR intervals and associated DUS signals with PSD and ApEn properties similar to those of normal and acidotic fetuses.
2) Estimating PSD and ApEn features.
3) Evaluating differences between fHRV features estimated from $FHR_{DUS}$ and $FHR_{RRI}$.
4) Evaluating the discriminability of different simulated fHRV features when applied to normal and acidotic signals.

### PhysioNet Fetal ECG Database

We used 80 $FHR_{RRI}$ tracings to validate our simulated RR intervals. These signals were acquired from two databases that included fetal ECG signals and reference annotations indicating the location of the QRS complexes. These annotations were provided by a mixture of experts, volunteers, and specialized algorithms. The first database was acquired by Jezewski et al. and published in PhysioNet (Goldberger et al., 2000; Jezewski et al., 2012). This database contains abdominal and direct fetal ECG records from five term fetuses (gestational ages 38–41 weeks), for 5 minutes each. The second database comprises 75 annotated fetal ECG recordings, each 1 minute long, utilized in the PhysioNet Computing in Cardiology Challenge 2013 (Goldberger et al., 2000; Silva et al., 2013). This database does not indicate the gestational age of the subjects, although the annotations were usually done using simultaneously acquired direct fECG signals. The application of direct fECG is only possible during labor after the rupture of the membranes. The databases do not indicate whether any the fetuses presented any pathological condition. Given the high incidence of normal fetuses, it is likely that the signals were acquired from normal fetuses. The databases also include the location of each R-wave. We used these locations to estimate RR intervals and extracted fHRV features from the RR intervals. We used these fHRV features to validate that our simulations were representative of real data.

### Simulation of $\mathrm{FH}R_{\mathrm{RRI}}$ and $\mathrm{FH}R_{\mathrm{DUS}}$

**FIGURE 1:**
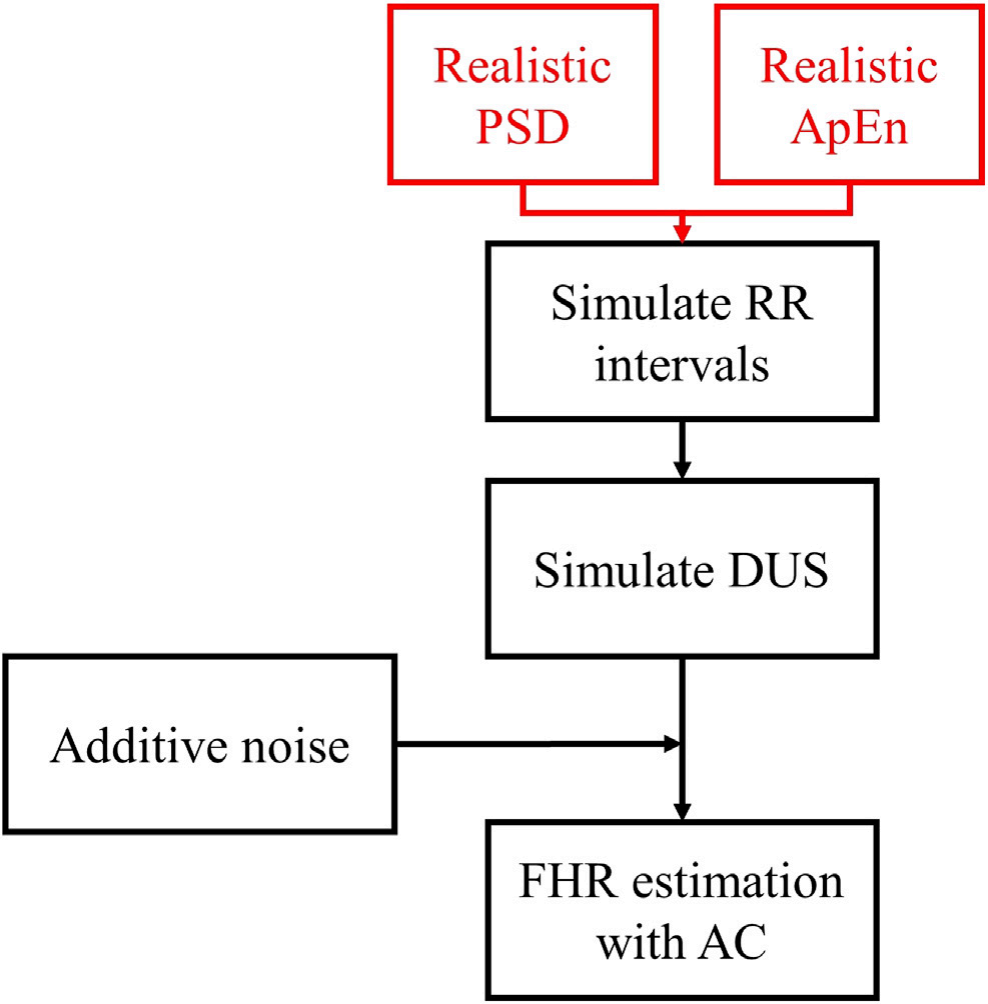
Diagram of the simulation of $FHR_{RRI}$ and $FHR_{DUS}$ signals. The PSD and ApEn distributions reported by Gonçalves et al. (2013) are used to generate random simulations of $FHR_{RRI}$ with similar fHRV. Then, we simulate DUS signals that correspond to the $FHR_{RRI}$ and we add noise. Finally, we use the AC to estimate the $FHR_{DUS}$.

Figure 1 outlines the process for simulating RR intervals, DUS signals, and uniformly sampled FHR. We first generated a sequence of random RR intervals with spectral features for normal or acidosis fetuses similar to those reported by Gonçalves et al., 2013. The 95% confidence intervals (CI) of the power in the low frequency, movement frequency, and high frequency bands reported by Gonçalves et al. are reported in Table 1. Afterwards, we generated the DUS envelope signals corresponding to the simulated RR intervals with added noise. Finally, we applied the AC method with a sliding window to generate uniformly sampled $FHR_{DUS}$.

**TABLE 1:** 95% Confidence intervals (CI) for the fHRV estimates reported by Gonçalves et al., 95% CI of the simulated RR intervals fHRV, and the difference of the limits of the 95% CI between the Normal and Acidosis distributions.

	Normal		Acidosis		Difference	
	Gonçalves	Simulated	Gonçalves	Simulated	Gonçalves	Simulated
	95% CI	95% CI	95% CI	95% CI		
LF	19.3	52.78	26.39	129.41	7.09	76.63
	77.21	86.52	264	231.13	186.79	144.61
MF	2.79	2.87	3.36	22.67	0.57	19.8
	13.60	18.96	54.77	110.20	41.17	91.24
HF	0.89	1.63	0.91	10.03	0.02	8.4
	2.25	21.3	8.09	23.35	5.84	2.05
LF/(MF + HF)	4.06	1.80	4.19	1.56	0.13	−0.24
	5.06	9.60	6.19	5.63	1.13	−3.97
ApEn	0.35	0.42	0.25	0.58	−0.1	0.16
	0.52	0.69	0.76	0.79	0.24	0.10

#### RR Interval Simulation

We simulated realizations of RR interval sequences, with controlled fHRV PSD structure and nonlinear complexity, as follows:

##### PSD

We first generated a continuous FHR signal, sampled at 4 Hz, with the desired fHRV spectrum. To do so, we filtered the same white Gaussian noise with three bandpass filters, corresponding to the three bands of interest for fHRV [from (Signorini et al., 2003)]: Low frequency (LF) 0.03–0.15 Hz; Movement frequency (MF) 0.15–0.5 Hz; and High frequency (HF) 0.5–1 Hz band. The three filter outputs were summed in different proportions to generate a signal whose spectrum matched the fHRV spectra reported by Gonçalves et al. (2013).

We then generated a continuous RR interval signal, $RR_{C}\left(t\right)$, from this FHR as $RR=\frac{60}{FHR}$, and upsampled it to 1 kHz using spline interpolation. However, the RR sequence is actually a point process in which the only information of interest is the time of occurrence of an event. Consequently, we transformed the continuous $RR_{C}\left(t\right)$ signal into a point process, $RR_{PP}\left[i\right]$, using the method of Clifford and Tarassenko (2005) which proceeds as follows:

1) Sample $RR_{C}\left(t\right)$ at time $t_{1}$. Its amplitude, $RR_{C}\left(t_{1}\right)$, determines the length of the first RR interval. Thus, $RR_{PP}\left[1\right]=RR_{C}\left(t_{1}\right)$. Find the value $RR_{C}\left(t_{2}\right)$, where $t_{2}\geq RR_{C}\left(t_{1}\right)+t_{1}$. Then, $RR_{PP}\left[2\right]=RR_{C}\left(t_{2}\right)$.
2) Repeat for the length of $RR_{C}\left(t\right)$. At each point $RR_{PP}\left[i\right]=RR_{C}\left(t_{i}\right)$ such that $t_{i}\geq RR_{C}\left(t_{i-1}\right)+t_{i-1}$.

The resulting $RR_{PP}\left[i\right]$ sequence was fitted to an Autoregressive (AR) model using the Yule-Walker method (“aryule” in the Matlab Signal Processing Toolbox). Multiple RR interval sequences were then generated by filtering independent realizations of white Gaussian noise with this AR model.

##### Approximate Entropy

ApEn is a measure of signal complexity, and thus random signals will have higher ApEn compared to periodic signals. To control the ApEn of our simulated RR intervals we modified the MIX process of Ferrario et al. (2006). The original MIX process switches randomly between a periodic signal and a uniformly distributed random signal and so does not permit the control of the realization’s PSD. To do so, we modified the MIX process to switch randomly between

1) A random sequence $RR_{r}\left[i\right]$, with the desired PSD, generated by filtering white noise with the RR AR model.
2) A semi-periodic signal $RR_{sp}\left[i\right]$ generated by concatenating segments of signal $RR_{r2}\left[i\right]$ with the desired PSD. Each segment has the same length l≥33 s and a randomly selected initial point $i_{1}\leq l*O_{f}$, where $O_{f}$ is the overlap factor. This will generate sequences with a limited number of patterns. Varying the overlap makes it possible to generate signals with different values of ApEn but the same PSD.

The MIX process switching is controlled by a binary random variable x, that will have a value of one with probability p, and zero otherwise. Varying p will change ApEn without changing the PSD. The i *i*th RR interval is generated by the MIX process as:

$$RR_{MIX}\left[i\right]=x\left[i\right]*RR_{sp}\left[i\right]+\left(1-x\left[i\right]\right)*RR_{r}\left[i\right]$$

#### DUS Envelope Simulation

Each RR interval sequence was transformed into a corresponding DUS envelope signal, sampled at 1 kHz, as follows:

1) A template DUS envelope cycle $DUS_{t}$ was selected randomly from 15 available periods of the DUS signal envelope shown in Hamelmann et al. (2020).
2) For each RR interval, the selected $DUS_{t}$ was stretched or contracted to a length equal to $RR_{MIX}\left[i\right]$ to give DUS(t,i).
3) Consecutive DUS(t,i) were concatenated to generate the DUS(t) signal.
4) A random additive noise signal, n(t), with a uniform distribution and a LF PSD was generated using an algorithm proposed by Nichols et al. (2010). We limited the power of the noise to 7.7 Hz, the same band of the envelope of the DUS(t) signal.
5) The amplitude of n(t) was varied along each realization to control the signal-to-noise ratio (SNR).
6) Finally, we generated $DUS^{'}\left(t\right)=DUS\left(t\right)+n\left(t\right)$.

**FIGURE 2:**
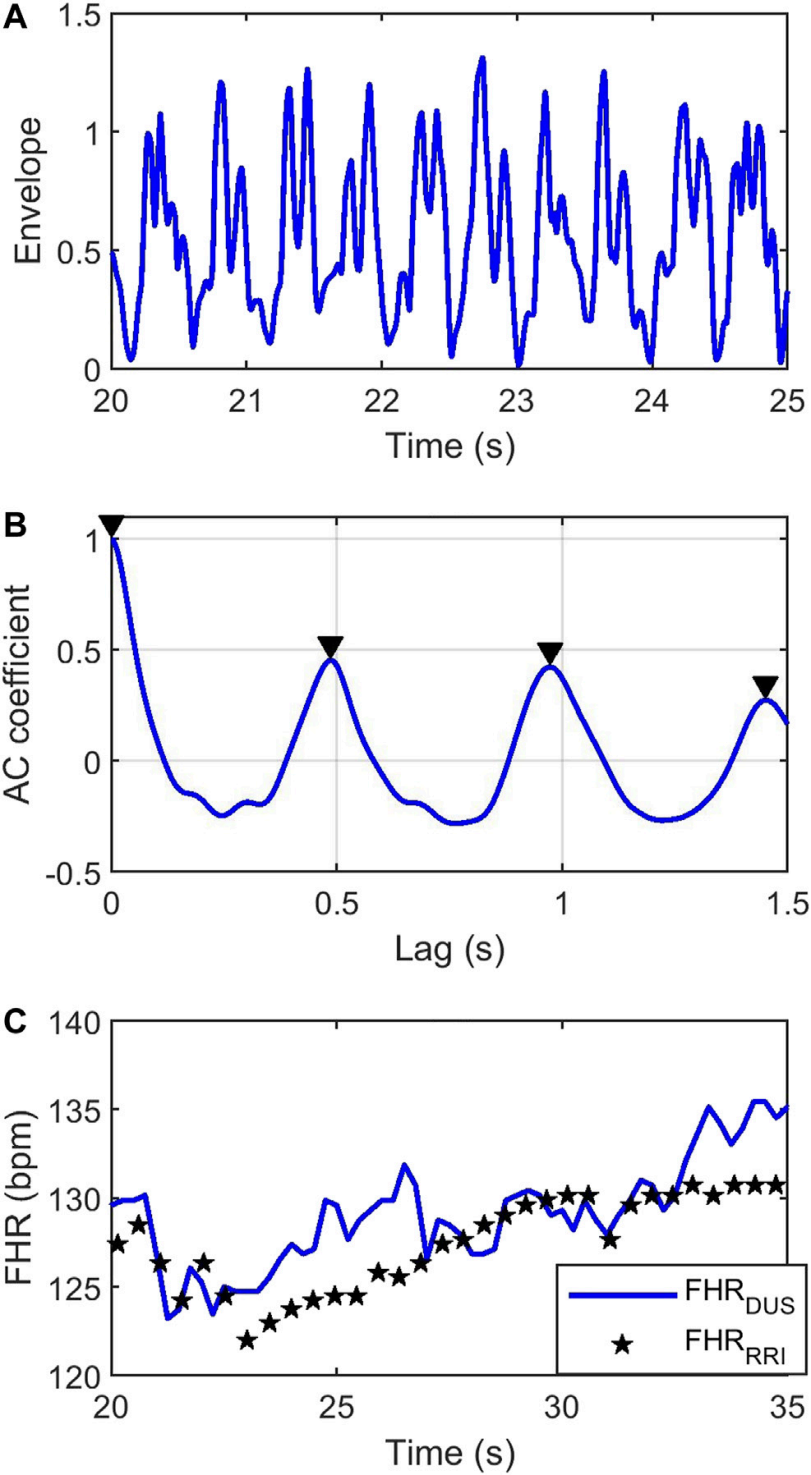
**(A)** Simulated envelope of the $DUS^{'}\left(t\right)$ signal using a series of $FHR_{RRI}$ extracted from the PhysioNet Database and 20 dB SNR. **(B)** AC coefficient (blue) and peaks (black triangles) of the DUS envelope using a 4 s window. **(C)** Simulated $FHR_{DUS}$ (blue), and the non-uniformly sampled $FHR_{RRI}$ (black stars).

Figure 2A shows a segment of a simulated $DUS^{'}\left(t\right)$ using the RR intervals from a subject in the PhysioNet Database and a SNR of 20 dB. Separate bursts of activity corresponding to cardiac cycles are apparent.

#### The AC Method

FHR was estimated from the DUS signal by computing its autocorrelation function (AC). The autocorrelation function of a periodic signal is also periodic with the same period. Consequently, the first non-zero maxima in the AC function will reflect the average RR interval. Figure 2B shows the AC coefficient function of the DUS signal in Figure 2A, estimated from a 4s window. The first non-zero-lag peak occurs at ∼0.5 s indicating an FHR = 120 bpm. Sliding the AC window across the signal with steps of 0.25 s will generate an FHR signal sampled at 4 Hz. The blue curve in Figure 2C shows the $FHR_{DUS}$ computed in this way from the signal in Figure 2A. (Note that Figure 2C covers a longer time span than Figure 2A). The black stars show the $FHR_{RRI}$ computed from the original RR intervals for comparison purposes. The AC estimates follow the trend of the $FHR_{RRI}$ but deviate around this trend due to the additive noise.

### fHRV Differences Between $\mathrm{FH}R_{\mathrm{RRI}}$ and $\mathrm{FH}R_{\mathrm{DUS}}$

**FIGURE 3:**
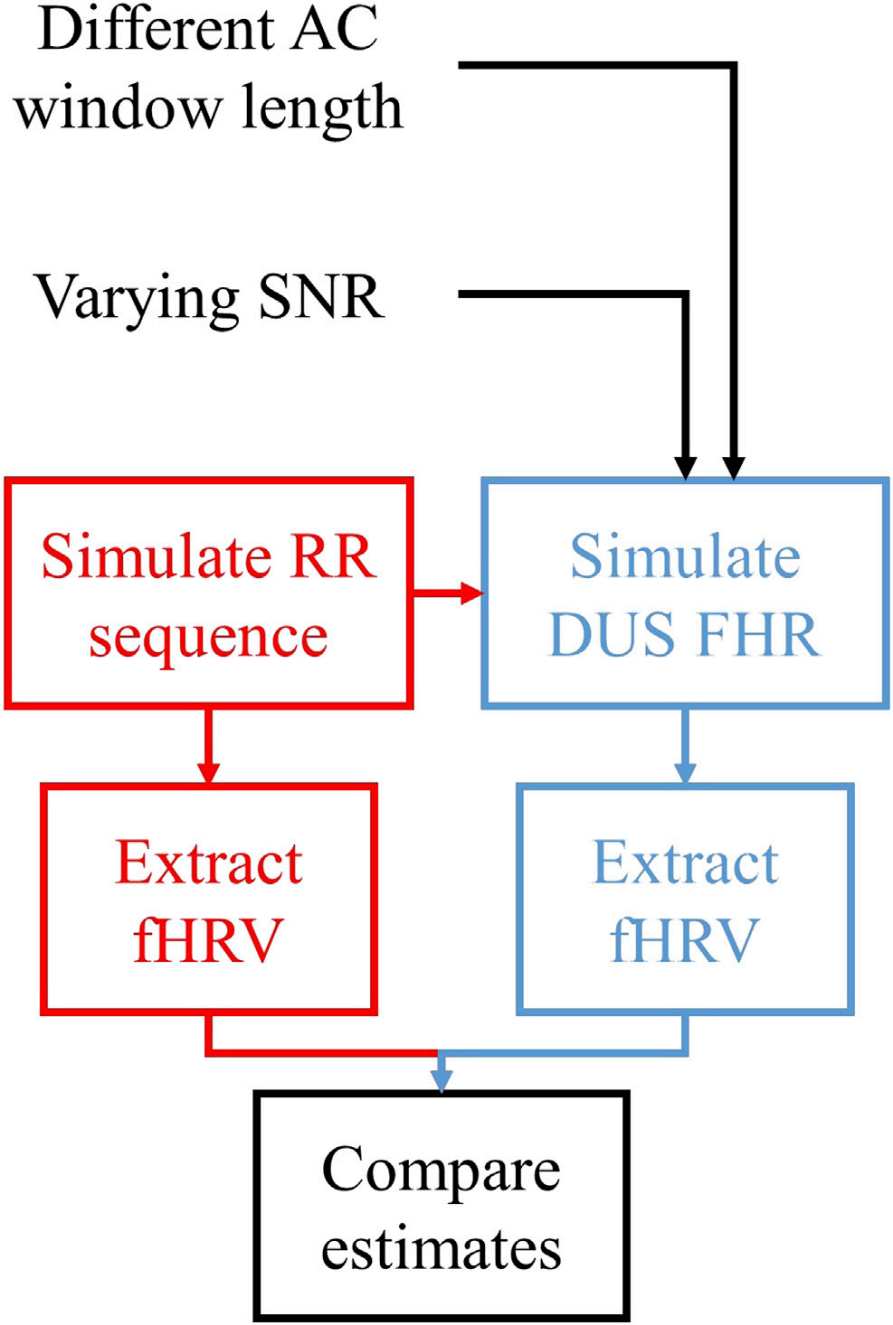
Outline of the assessment of the fHRV estimation differences between $FHR_{RRI}$ and $FHR_{DUS}$. We simulate a set of RR intervals, and estimate the fHRV features. We also use these sequences to simulate $FHR_{DUS}$ varying AC window length and SNR. Finally, we estimate fHRV from these signals and compare the differences in estimates from $FHR_{RRI}$ and $FHR_{DUS}$.

Figure 3 shows the procedure used to compare the fHRV estimates from the RR intervals and DUS FHR.

1) $RR_{MIX}\left[i\right]$ sequences were generated with fHRV distributions similar to those reported by Gonçalves et al. for normal and acidotic fetuses.
2) These $RR_{MIX}\left[i\right]$ sequences were then used to generate corresponding $FHR_{DUS}$ signals and $FHR_{RRI}=\frac{60}{RR_{MIX}}$.
3) fHRV estimates were obtained from $FHR_{DUS}$ and $FHR_{RRI}$.
4) The estimates were compared as a function of AC window length and SNR.

We simulated 1,000 Monte Carlo (MC) $FHR_{RRI}$ signals having normal and acidotic properties. This yielded a total of 2,000 $FHR_{RRI}$. For each realization of $FHR_{RRI}$ we generated $DUS^{'}\left(t\right)$ signals with 21 SNR values (ranging from −10 to 30 dB in 2 dB steps). These signals were then transformed into $FHR_{DUS}$, as described above, using 17 AC window lengths (ranging from 1 to 5 in 0.25 s steps). This resulted in 714,000 $FHR_{DUS}$ signals.

#### FHR Preprocessing

The $FHR_{DUS}$ signals were preprocessed before estimating fHRV features. In some cycles, the additive noise in the DUS signal prevented the peak-finding algorithm from finding the peak that corresponded to the average FHR. To reduce the effect of these outliers, we estimated the moving median of $FHR_{DUS}$ over a 5s window. Estimates that deviated more than 40 bpm from the moving median were removed and replaced by linear interpolation of the adjacent samples. Finally, we limited the estimated $FHR_{DUS}$ to a range of 60–180 bpm.

#### fHRV Features

The PSDs of the non-uniformly sampled $FHR_{RRI}$, and the uniformly sampled $FHR_{DUS}$ signals were estimated using the Lomb-Scargle (LS) periodogram as implemented in the function “plomb” in the Matlab Signal Processing Toolbox. We chose the LS periodogram, since it provides unbiased estimates of the power spectrum in non-uniformly sampled signals (Clifford and Tarassenko, 2005). Using alternative methods, such as the Welch periodogram or AR models, would require resampling $FHR_{RRI}$ to a continuous signal, which leads to a biased estimate of the power spectrum (Clifford and Tarassenko, 2005). The normalized power in three frequency bands was then computed as:

$$LF_{pow}=\frac{\sum_{0.03}^{0.15}LS\left(f\right)\Delta f}{\sum_{0.03}^{1}LS\left(f\right)\Delta f}$$

$$MF_{pow}=\frac{\sum_{0.15}^{0.5}LS\left(f\right)\Delta f}{\sum_{0.03}^{1}LS\left(f\right)\Delta f}$$

$$HF_{pow}=\frac{\sum_{0.5}^{1}LS\left(f\right)\Delta f}{\sum_{0.03}^{1}LS\left(f\right)\Delta f}$$

where LS(f) is the PSD estimated using the LS periodogram. In addition, we estimated the LF/(MF + HF) ratio.

ApEn, a measure of the nonlinear complexity of FHR, was estimated as follows:

1) FHR was decimated to 2 Hz, following Gonçalves et al., 2013 who found that sampling the FHR at 2 Hz provided better ApEn estimates than 4 Hz.
2) The function “approximateEntropy” in the Matlab Predictive Maintenance Toolbox was used with an embedding dimension of 2, and radius of 0.2.

#### Feature Comparison

Differences between features computed from the RR and DUS signals were quantified in terms of their bias and random differences:

$$bd=\frac{E\left[f_{DUS}\right]-E\left[f_{RRI}\right]}{E\left[f_{RRI}\right]}*100\%$$

$$rd=\frac{\sqrt{\sum {\left(f_{DUS}-f_{RRI}\right)}^{2}}}{E\left[f_{RRI}\right]}*100\%$$

where bd and rd are the normalized bias and random differences, $f_{DUS}$ is a feature estimated from $FHR_{DUS}$, $f_{RRI}$ is a feature estimated from $FHR_{RRI}$ and E[x] is the expected value.

### Discriminability of fHRV

**FIGURE 4:**
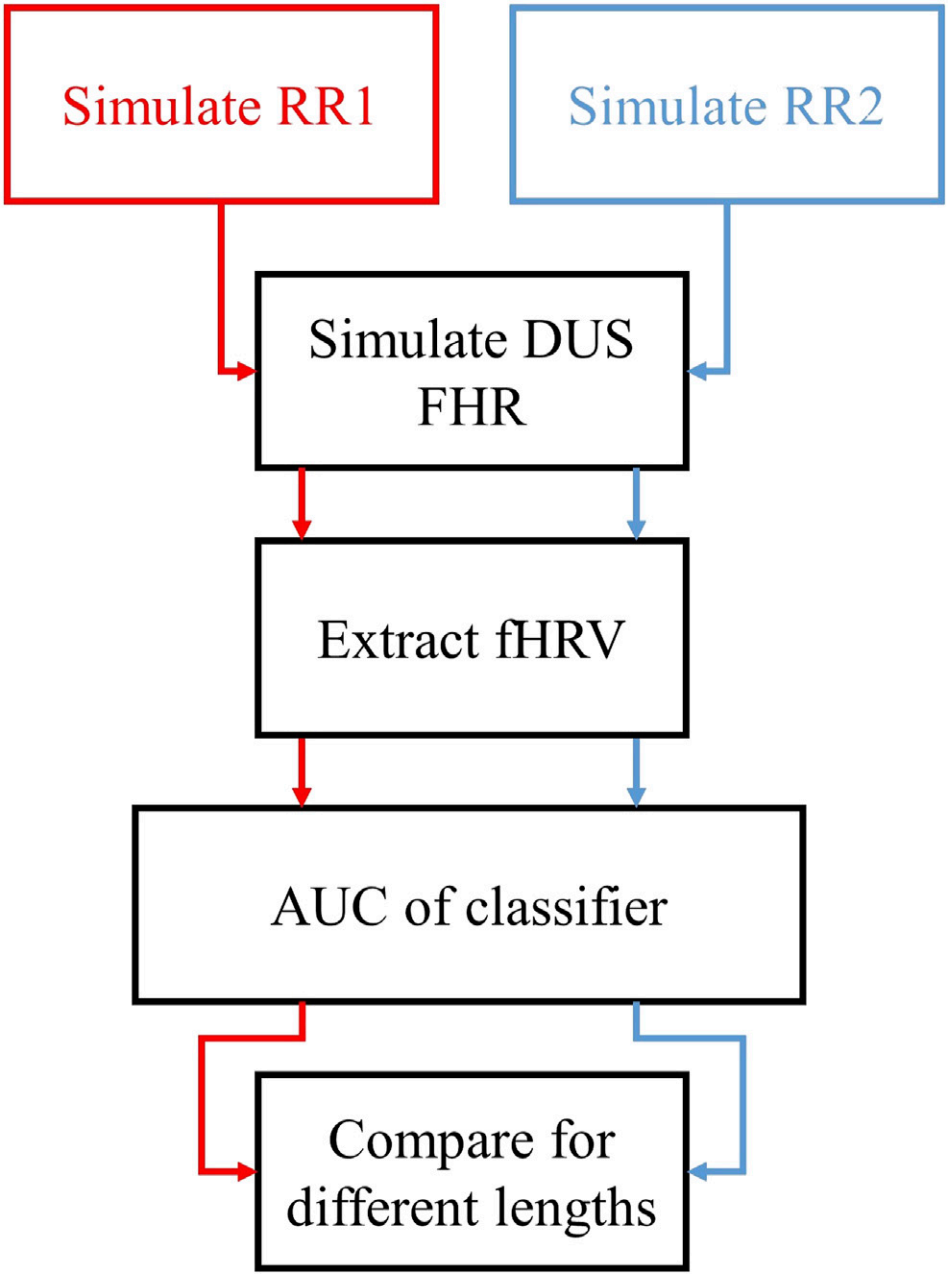
Outline of the assessment of the discriminability of fHRV features as functions of the AC window length. We simulated two sets of $FHR_{RRI}$ sequences with different PSD and ApEn distributions. Then, we extracted the fHRV of the simulated $FHR_{DUS}$ for each case, and we used these estimates to assess the discriminability of each feature using the AUC of Neyman-Pearson classifiers.

Figure 4 describes the procedure used to assess fHRV discriminability. We simulated normal and acidotic $FHR_{RRI}$. To remove the effect of the signal amplitude, each realization of $FHR_{RRI}$ was scaled to have a standard deviation of 21.63 bpm, midway between the two reported distributions. Then $FHR_{DUS}$ signals were generated for each $FHR_{RRI}$ realization. PSD features and ApEn were computed for the RR and DUS signals. We constructed a Neyman-Pearson classifier for each signal that used the likelihood ratio of the normal and acidosis distributions, and we estimated the area under the curve (AUC) for the $FHR_{RRI}$ and $FHR_{DUS}$ realizations. The AUC and 95% confidence intervals (CI) were estimated from 1,000 bootstrap samples of the normal and acidotic distributions. To compare features, we computed the following metrics:

1) $AUC_{RRI}$ is the median of the 1000 AUC estimates obtained from bootstrap sampling each pair of fHRV distributions estimated from $FHR_{RRI}$. $AUC_{DUS}\left(wl,SNR\right)$ is the median of the 1000 AUC estimates obtained from bootstrap sampling each pair of fHRV distributions as a function of the window length wl, and the SNR.
2) The normalized difference between $AUC_{RRI}$ and $AUC_{DUS}\left(wl,SNR\right)$ given as follows:

$$D_{AUC}=E\left[\frac{AUC_{RRI}-AUC_{DUS}\left(wl,SNR\right)}{AUC_{RRI}}\right]\;$$

where $D_{AUC}$ is the normalized difference, and $AUC_{DUS}\left(wl,SNR\right)$ is the $AUC_{DUS}$ as function of AC window length and SNR. $D_{AUC}$ quantifies the mean variation in the discriminability of each feature across all simulated window lengths and SNRs.

3) The normalized standard deviation of $AUC_{DUS}$ for all lengths and SNR given as follows:

$$\sigma_{AUC}=\frac{\sqrt{E\left[{\left(AUC_{DUS}\left(wl,SNR\right)-E\left[AUC_{DUS}\right]\right)}^{2}\right]}}{AUC_{RRI}}$$

where $\sigma_{AUC}$ is the normalized standard deviation. This metric quantifies how the discriminability of each feature varies as the window length and SNR vary.

## Results

### Simulation of $\mathrm{FH}R_{\mathrm{RRI}}$ and $\mathrm{FH}R_{\mathrm{DUS}}$

We first compared the features of the simulated $FHR_{RRI}$ sequences to those reported by Gonçalves et al. (2013) for fetuses with normal umbilical cord blood-gas pH (≥7.20) and those with acidotic pH (<7.20). Table 1 compares the 95% confidence intervals of the PSD and ApEn features reported by Gonçalves et al. to those estimated from our simulated sequences. Table 1 also shows the difference in the limits of the acidosis and normal distributions. Although the absolute limits of the simulated distributions differ from the reported distributions, they have similar trends; all features except for the LF/(MF + HF) ratio, are larger for the acidosis than the normal class.

**FIGURE 5:**
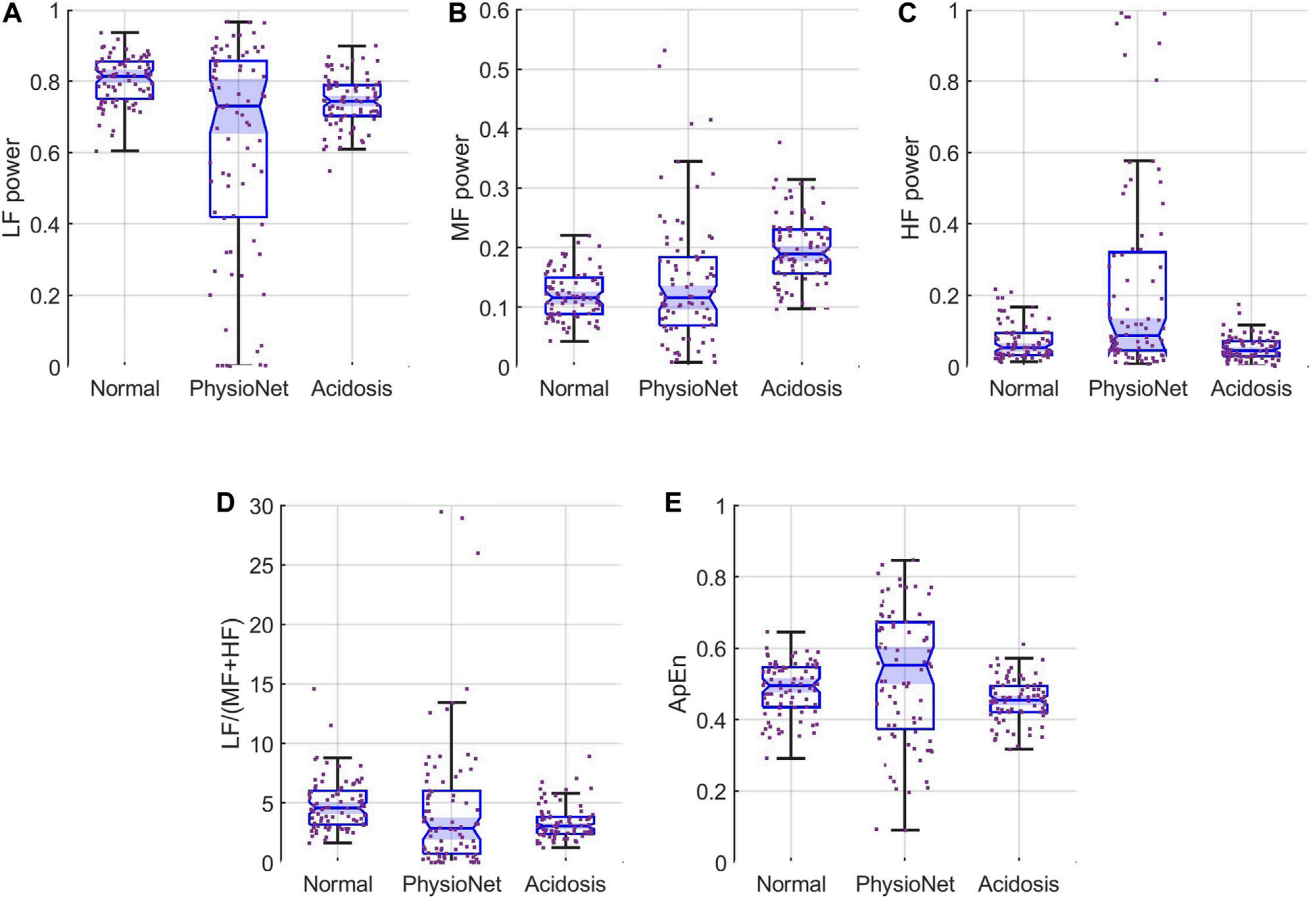
Feature distributions for **(A)** LF, **(B)** MF, and **(C)** HF **(D)** LF/(MF + HF) ratio, and **(E)** ApEn. Each panel show the samples (scattered points) and boxplots for the features for simulated normal (left), the PhysioNet data (middle), and simulated acidosis $FHR_{RRI}$ features (right). The notches, or indentations, in each boxplot indicate the 95% CI for the median of each distribution. These plots show that the PhysioNet data and the simulated normal are not significantly different for any of the features. In contrast, the simulated acidosis distributions are significantly different for the MF and ApEn.

We also compared the features of our simulated sequences to those of the 80 subjects in the PhysioNet database. Figure 5 shows boxplots of the normalized $FHR_{RRI}$ features for three populations: simulated normal (left), PhysioNet data, and simulated acidosis (right). The notches, or indentations, in the box plots indicate the 95% CI of the median of each distribution. From these, it is evident that the medians obtained of the PhysioNet subjects and the simulated normal sequences were not statistically different for any feature. The 95% CI of the medians from the 80 subjects also overlap those of the acidosis $FHR_{RRI}$ except for the MF power and the ApEn. Thus, the distribution of the fHRV features estimated from our simulated $FHR_{RRI}$ were similar to those of real data.

### fHRV Differences Between $\mathrm{FH}R_{\mathrm{RRI}}$ and $\mathrm{FH}R_{\mathrm{DUS}}$

**FIGURE 6:**
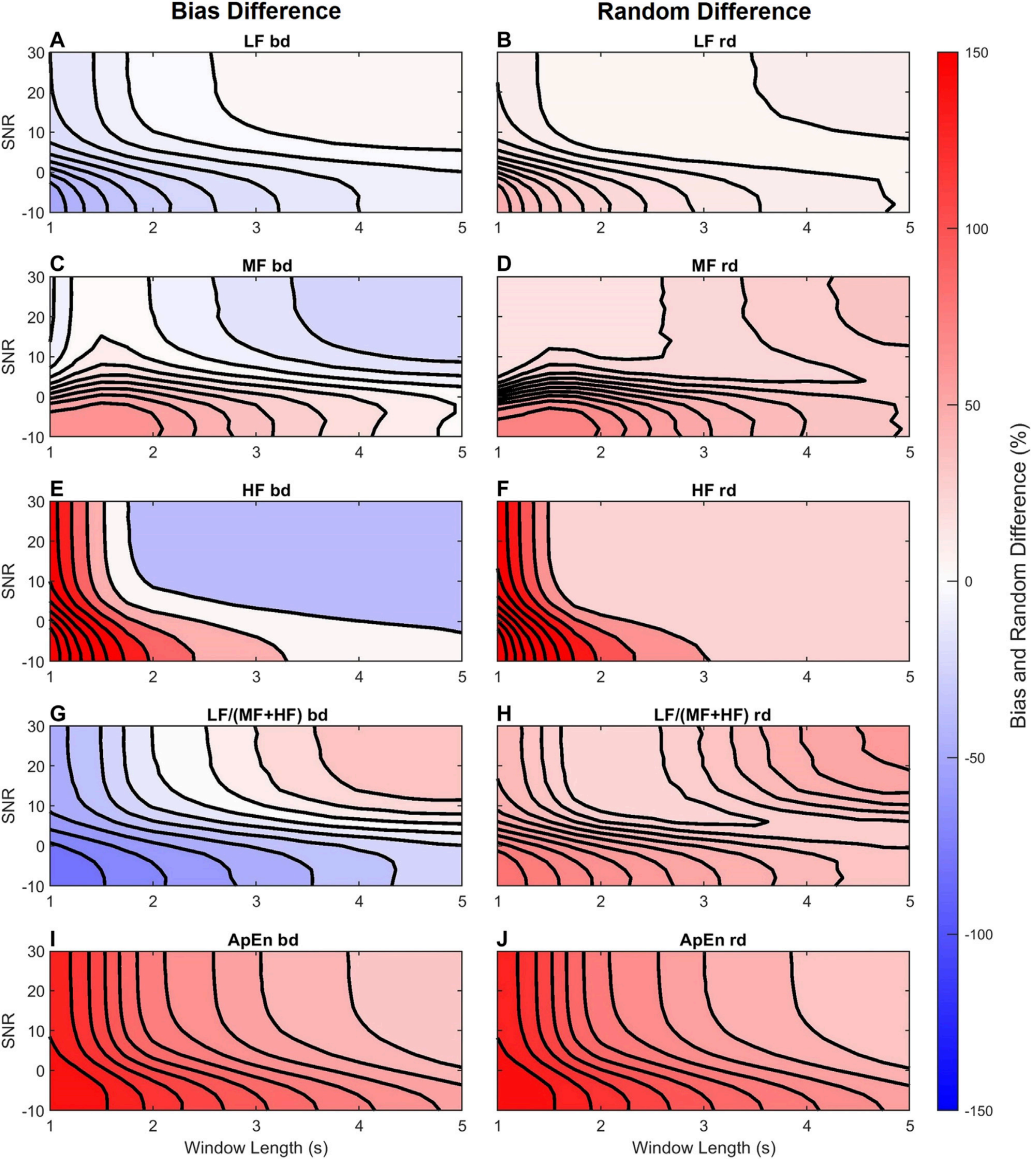
Contour plots of the bias difference, bd, and random difference, rd, of the LF **(A,B)**, MF **(C,D)**, HF **(E,F)**, LF/(MF + HF) **(G,H)**, and ApEn **(I,J)** features from $FHR_{DUS}$ for the acidosis distributions and varying AC window length (horizontal axis) and SNR (vertical axis). The differences are coded in colors blue (negative), white (zero), and red (positive) according to their magnitude. For visualization, 10 isolines are used in each panel.

Next we examined the differences between features computed from the simulated $FHR_{RRI}$ and $FHR_{DUS}$. Figure 6 shows contour plots for the bias (left column) and random (right column) differences of the five features as functions of window length and SNR. The magnitude of the differences are color coded from blue (negative) to white (zero) and to red (positive). Figure 6A shows the LF power is underestimated when the SNR is low or the window length is short; the bias difference is close to zero difference when the window length is longer than 2 s and the SNR is greater than 10 dB. Figure 6B shows that the random difference of LF behaved similarly; variability was higher for low SNR and short AC window lengths and decreased as either parameter increased.

Figures 6C,E show that the MF and HF powers were overestimated for low SNR and short AC windows while for long windows and high SNRs they were underestimated. The bias differences had larger magnitude for HF than for MF. Thus, the HF power was most sensitive to the AC window and additive noise. Figures 6D,F show that the random difference for both MF and HF were larger for low SNR and short AC windows but decreased as the SNR and window length increased.

Figures 6G,H show that LF/(MF + HF) ratio bd and rd behaves as expected from the individual trends. Thus, for low SNR and short windows, the ratio was underestimated: smaller LF divided by larger MF and HF estimates produce an underestimated ratio. Similarly, for longer windows and higher SNR, the ratio was overestimated; an almost unbiased LF divided by smaller MF and HF produce an overestimate. The random difference in Figure 6H is more complicated to interpret. It was higher for low SNR and short windows, and decreased as either parameter increased. However, it reached a minima at an SNR of 10 dB and window length of 2.5 s and then increased for higher SNRs and window lengths. This might be explained if we consider that the denominator of this ratio (MF + HF) is underestimated in this area. Thus, any variability in the LF estimate, divided by a smaller estimate of (MF + HF) will yield a more variable estimate.

Figure 6I shows that ApEn was always overestimated with the error decreasing as the SNR and window length increased. The random error of ApEn (Figure 6J) behaved similarly.

### Discriminability of fHRV

**FIGURE 7:**
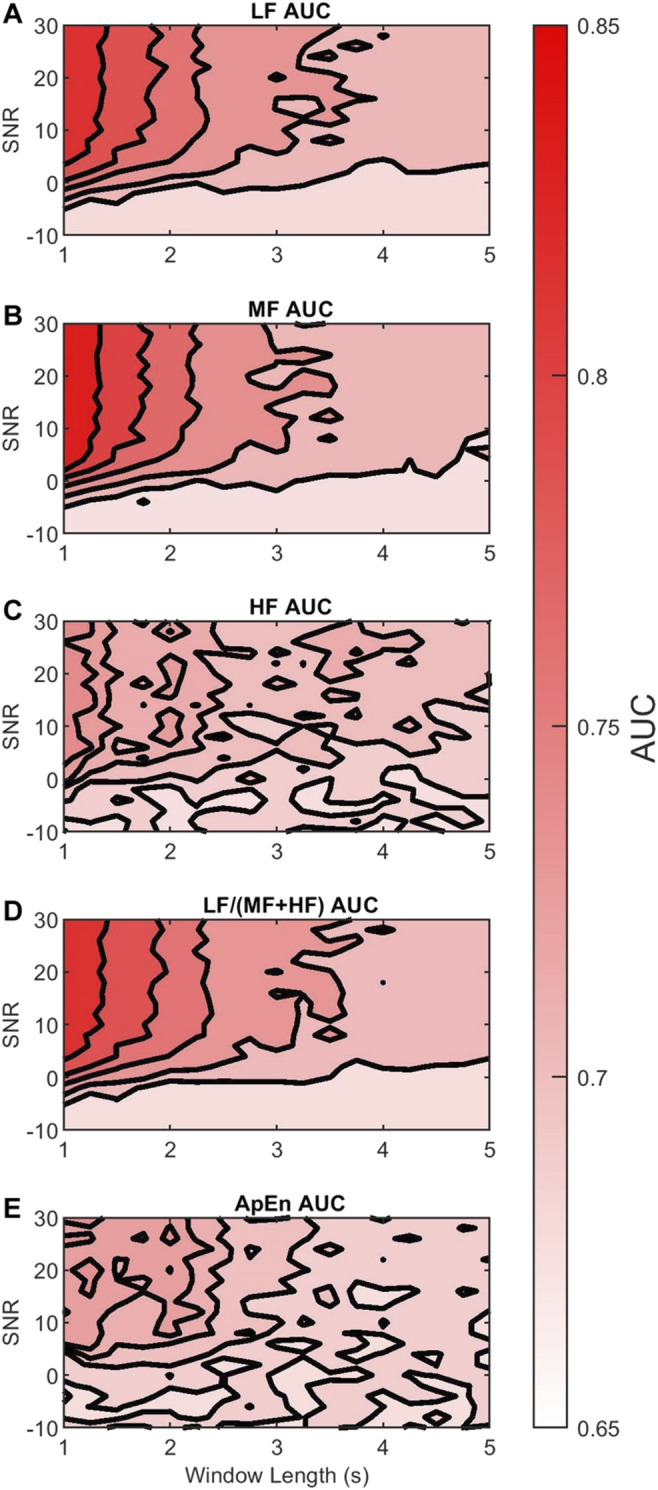
Contour plots of the median $AUC_{DUS}$ of the **(A)** LF, **(B)** MF, **(C)** HF, **(D)** LF/(MF + HF), and **(E)** ApEn features for varying AC window length (horizontal axis) and SNR (vertical axis). The $AUC_{DUS}$ are coded in colors white (0.65), and red (0.85) according to their magnitude. For visualization, 5 isolines are used in each panel.

We evaluated the discriminability of the features in terms of the AUC of the Neyman-Pearson classifiers for varying AC window length and SNR. As a reference, Table 2 shows the median and 95% CI $AUC_{RRI}$. Figure 7 shows in contour plots the $AUC_{DUS}$ of each of the LF, MF, HF, LF/(MF + HF), and ApEn features. For all cases, $AUC_{DUS}$ decreased with respect to $AUC_{RRI}$ due to the AC method and additive noise. The plots are color coded from the minimum $AUC_{DUS}=0.65$ (white) to a maximum $AUC_{DUS}=0.85$ (red). Figure 7 describes two main trends: 1) for all features, $AUC_{DUS}$ decreases as the SNR decreases, and 2) for all features, $AUC_{DUS}$ decreases as the AC window length increases.

**TABLE 2:** Median $AUC_{RRI}$ and 95% confidence intervals.

	$AUC_{RRI}$
$LF_{pow}$	0.83
	(0.81–0.84)
$MF_{pow}$	0.86
	(0.84–0.87)
$HF_{pow}$	0.82
	(0.80–0.83)
$\frac{LF}{MF+HF}$	0.82
	(0.80–0.84)
ApEn	0.92
	(0.91–0.93)

Figures 7A,B show that the discriminability of LF and MF was greatest for SNR larger than 0 dB and windows shorter than 2 s. Thus, in this region, their discriminability was not affected much by the AC method. However, outside this region the color contrast is strong, indicating a large drop in discriminability. In contrast, Figure 7C shows that the reduction in HF discriminability was less marked as the window length increased or SNR decreased.

These observations can be contrasted with the results in Table 3. Table 3 shows the 95% CI of the differences between $AUC_{RRI}$ and $AUC_{DUS}$, the mean difference $D_{AUC}$, and $\sigma_{AUC}$ for each feature, AC window 1–5 s long, and −10–30 dB SNR. The $D_{AUC}$ estimates show that in average the LF power loses 10.99%, MF power loses 14.22%, and HF power loses 13.32% of their discriminability. However, the variability of this discriminability, according to $\sigma_{AUC}$, is considerably higher for LF and MF (5.02 and 5.40%) than for HF (2.05%). Thus, although HF loses 13.32% of its discriminability due to the AC method and additive noise, the obtained discriminability only varies in 2.05% with respect to the AC window length and the SNR.

**TABLE 3:** Mean, $D_{AUC}$, and 95% CI of the difference between $AUC_{RRI}$ and $AUC_{DUS}$ and standard deviation of the estimated $AUC_{DUS}$, $\sigma_{AUC}$, for AC windows 1–5 s long and −10–30 dB SNR.

	Mean difference $D_{AUC}$ (%) and 95% CI	Standard deviation $\sigma_{AUC}$ (%)
$LF_{pow}$	−10.99 (−17.29–1.25)	5.02
$MF_{pow}$	−14.23 (−20.92–−0.40)	5.40
$HF_{pow}$	−13.33 (−16.39–−8.38)	2.05
$\frac{LF}{MF+HF}$	−10.39 (−16.82–−2.14)	5.15
ApEn	−24.17 (−26.30–−20.18)	1.76

Figure 7D shows that the discriminability of LF/(MF + HF) ratio decreases with longer windows and lower SNR. It follows a similar trend to the LF and MF. The estimated D_AUC showed a decrease of 10.39% of its discriminability, and $\sigma_{AUC}$ showed a variability of 5.15%. These estimates were close to those of the LF power.

Finally, Figure 7E shows that the discriminability of ApEn behaved similarly to HF $AUC_{DUS}$. In this case, $D_{AUC}$ showed the largest loss of discriminability (24.17%) but the smallest $\sigma_{AUC}$ variability (1.76%). This means that although ApEn loses much of its discriminability due to the AC method, the remaining discriminatory information is affected little by varying SNR or AC window lengths.

## Discussion

This paper has two objectives: 1) to analyze differences in fHRV features estimated from $FHR_{RRI}$ and $FHR_{DUS}$; and 2) to determine how these differences influenced their ability to discriminate between two fHRV distributions. In our analysis, we simulated sequences of RR intervals for which we controlled the PSD and ApEn. Then, we simulated the DUS sampling and AC method, and extracted the relevant features for each objective. Our results indicate that 1) our simulated $FHR_{RRI}$ sequences have fHRV features with distributions similar to those of real data, 2) the estimation of HF power and ApEn are the most affected by the AC method and additive noise, and 3) the loss of discriminability due to the AC method is largest for the ApEn and smallest for the LF power and LF/(MF + HF) ratio. We discuss below each section of these results.

### Simulation Issues

The results presented in this paper are based on simulations in which we generated artificial RR intervals and the corresponding DUS signals. The significance of our results will depend on the validity of these simulations. We believe they are valid for the following reasons:

First, an important feature of our simulation of RR intervals was that we were able to generate sequences having both power spectral and entropy features similar to those of real data. Table 1 and Figure 5 show that the distribution of fHRV features of our $FHR_{RRI}$ simulations fall within the distributions estimated from the available real data. All features of the simulated RR intervals were comparable to the features estimated from clinical data. This contrasts with previous simulations which controlled only the PSD (Clifford and Tarassenko, 2005), or the entropy independently (Ferrario et al., 2006).

Secondly, we opted to simulate the envelope of the DUS signals rather than the raw DUS signal itself. Raw DUS signals are subject to multiple artifacts during clinical acquisition: movement of the probe or signal loss introduce noise in the signals (Shakespeare et al., 2001; Jezewski et al., 2017). As a solution, fetal monitors use the envelope of the signal, which serves as a LF filter (Hamelmann et al., 2020). This envelope trades the amount of information contained in the signal, such as the location of specific cardiac events (Shakespeare et al., 2001), for robustness in the estimation of the FHR (Hamelmann et al., 2020). Investigating the effect of extracting the envelope of the signal is out of the scope of this study as we focused specifically on the AC method applied to the DUS envelope. Furthermore, we introduced noise in our signal in two ways: 1) we use 15 DUS envelopes reported in the literature (Hamelmann et al., 2020) as templates, which have intrinsic acquisition noise, and 2) we added bandlimited uniform noise, where the cut-off frequency was set to 7.7 Hz. Thus, even for our simulations with the highest SNR, there is noise inherent to the templates that cannot be removed. This introduces heterogeneity in the signal, each DUS cycle is different from the others.

Finally, it is important to consider the method used to compute $FHR_{DUS}$ from the DUS signal: the estimation of fHRV features will depend on this. We chose to use a standard AC estimation method since this is what is currently used in clinical monitors. Thus, our results are directly relevant to understanding how the properties acquired with current monitors behave. We are aware that a number of more sophisticated methods have been proposed to improve the accuracy of the beat-to-beat estimation of the FHR. These methods are based on the AC method with adaptive parameters, or utilize ML models to extract the beat-to-beat sequence from the DUS signal (Peters et al., 2004; Jezewski et al., 2011; Alnuaimi et al., 2017; Valderrama et al., 2019; Katebi et al., 2020). The effect of those methods on fHRV as function of their parameters is an important question to be explored but is beyond the scope of the present paper.

### fHRV Differences Between $\mathrm{FH}R_{\mathrm{RRI}}$ and $\mathrm{FH}R_{\mathrm{DUS}}$

Our experiments aimed to analyze the error of fHRV feature estimation from using the AC method. We found that the length of the autocorrelation window, which determines the extent of signal averaging, had a strong influence on these errors. Longer windows provide more AC averaging, which reduces the effect of additive noise at the cost of beat-to-beat accuracy in the estimation of FHR. In other words, longer averaging windows act as low-pass filters with lower bandwidths. Accordingly, Figure 6A shows underestimation of the LF power for short AC windows and low SNR, but the bias difference increases to be almost zero as AC window length and SNR increase. In contrast, the MF and HF powers were increasingly attenuated as the window length increased. As expected, the AC method attenuates the MF and HF power while increasing the relative magnitude of the LF power. This is in agreement with the findings of Clifford and Tarassenko (2005) which showed that interpolated heart rate signals (without averaging) overestimate LF power with respect to higher frequency bands.

Showing a different behavior, ApEn (Figure 6I) is always overestimated, which might be due to the effect of additive noise. The ApEn is an estimate of a signal irregularity, and it is higher for random than for periodic signals. Thus, adding random noise increases the signal irregularity which directly increases the ApEn. However, Figure 6I shows a decrease when SNR or the window length increase; less noise or more averaging reduces the irregularity in the signal and lowers the ApEn.

In summary, these results show that data from multiple monitors with different parameters may yield different estimates of fHRV. The extent of these differences is documented in our contour plots as a function of window lengths and SNR. Unfortunately, information about the window length used is rarely available for commercial monitors. Unless the manufacturers start to disclose the parameters of their acquisition algorithms, data analysis of such signals must take into account that the variability in the estimated fHRV does not only depend on fetal state but also the CTG monitor.

### Discriminability of fHRV

FHR monitoring during the intrapartum aims to detect fetuses at risk and to use this information to determine whether an emergency cesarean delivery is warranted. Thus, it is important to study how discriminability of certain features is affected by the CTG acquisition methods. Our simulations showed that the discriminability of PSD and ApEn features changed with AC window length and SNR. For all features, the AUC of a Neyman-Pearson classifier decreased as the SNR decreases. This is explained by loss of discriminatory information due to additive noise or large magnitude. Similarly, the AUC decreased as the AC window length increased. This is explained by the loss of discriminatory beat-to-beat information associated with longer AC windows (more averaging).

Two different behaviors can be observed for the five fHRV features analyzed. LF, MF, and LF/(MF + HF) lose less discriminability on average as defined by $D_{AUC}$. However, they have higher $\sigma_{AUC}$ variability across the whole range of AC window lengths and SNRs. This means that under ideal conditions (short AC windows and high SNR) the discriminatory information in these features is well preserved by the AC method. However, samples obtained from monitors that use different AC window lengths could carry quite different discriminatory information, as $\sigma_{AUC}$ is higher. In contrast, the HF and ApEn features lose more $D_{AUC}$ discriminability on average, but show less $\sigma_{AUC}$ variability. Thus, these features are more affected by the AC method itself, but they are less dependent on the parameters of this method. This means that when considering data from multiple sources, the LF, MF, and LF/(MF + HF) features might be the best discriminatory parameters if the data is analyzed for each source independently. However, if the data is mixed, then the HF and ApEn features will have the similar discriminatory behavior regardless the source.

**FIGURE 8:**
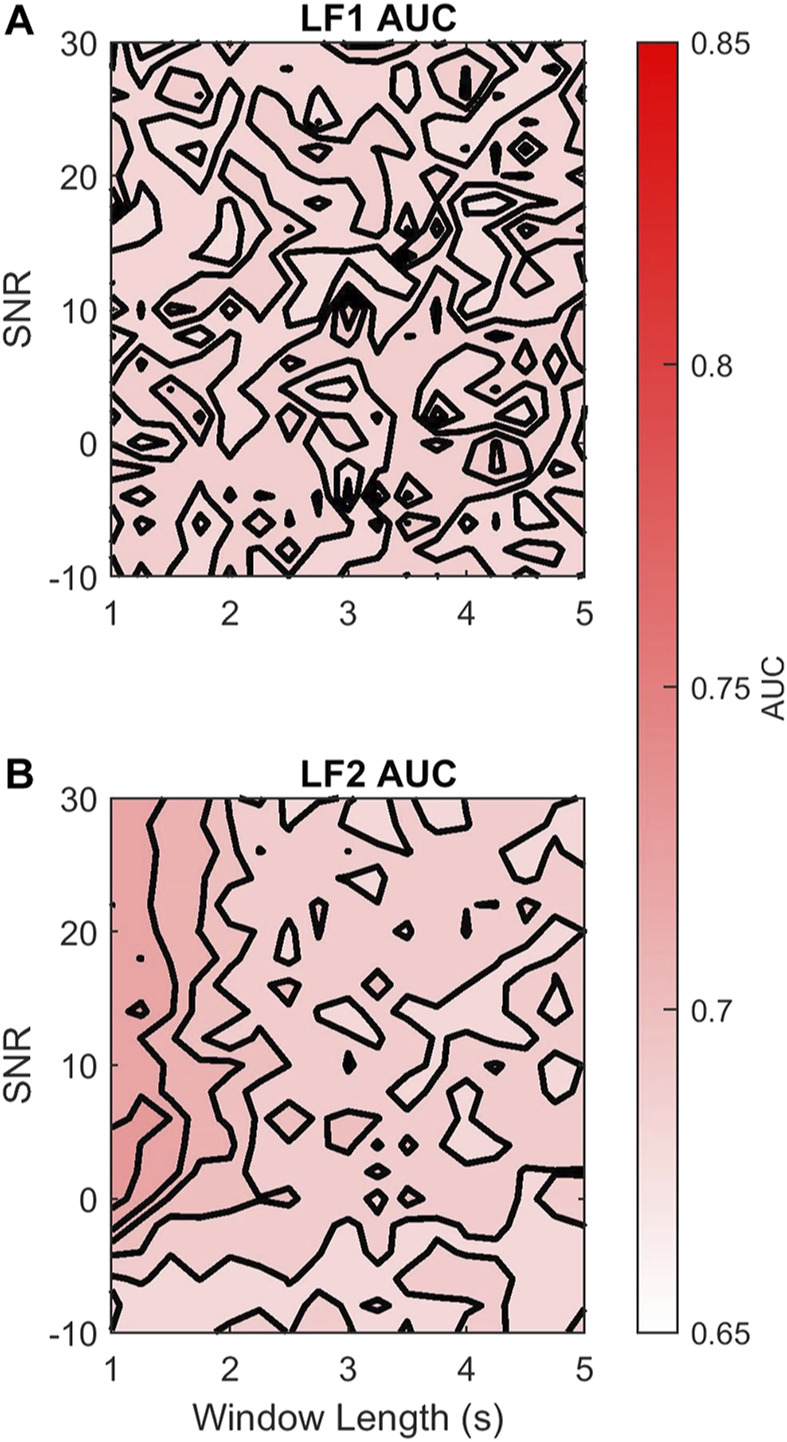
Contour plots of the median $AUC_{DUS}$ of the **(A)** LF1 and **(B)** LF2 sub-bands for varying AC window length (horizontal axis) and SNR (vertical axis). The $AUC_{DUS}$ are coded in colors white (0.65), and red (0.85) according to their magnitude. For visualization, 5 isolines are used in each panel.

The AC method is expected to preserve low frequencies and attenuate high frequencies. Thus, we hypothesized that there might be an LF sub-band that the AC method would not affect. To this end we explored the effects of dividing the LF band into two sub-bands LF1 (0.03–0.072 Hz) and LF2 (0.072–0.15 Hz). Figure 8A shows that by doing so, there was no loss in discriminability for LF1 across different AC window lengths and SNR. In contrast, Figure 8B shows that the discriminability of LF2 $AUC_{DUS}$ decreased with longer AC windows and lower SNR. Table 4 quantifies these changes; LF1 loses 0.02% of its $D_{AUC}$ discriminability, and has 0.74% of $\sigma_{AUC}$ variability across all the range of AC window lengths and SNRs. In contrast, LF2 loses 3.38% of its $D_{AUC}$ discriminability and has 1.74% of $\sigma_{AUC}$ variability. These results show that the use of the AC method reduces the discriminability of higher frequencies but that frequencies below 72 mHz are not affected by the acquisition method. Therefore, the power in these frequencies provides a discriminatory feature that is independent of the acquisition method and its parameters (AC window length and SNR).

**TABLE 4:** Median $AUC_{RRI}$ and 95% CI, mean difference of $AUC_{RRI}$ and $AUC_{DUS}$, $D_{AUC}$, and standard deviation of the estimated $AUC_{DUS}$, $\sigma_{AUC}$, for LF1 and LF2. These estimates were done for AC windows 1 to 5 s long and −10 to 30 dB SNR.

	$AUC_{RRI}$	Mean difference $D_{AUC}\;$ (%)	Standard deviation $\sigma_{AUC}$ (%)
LF1	0.69 (0.67–0.70)	−0.02 (−1.40–1.49)	0.75
LF2	0.72 (0.70–0.74)	−3.38 (−5.48–1.04)	1.74

### Limitations

We believe these results provide important insight into the effects of computing FHR features using the AC method. Nevertheless there are a number of limitations of the work to consider.

First, the reference distributions that we used to define the normal and acidotic classes were estimated from a handful of cases, which might not be enough. Gonçalves et al. (2013) reported features extracted from 21 normal fetuses and six acidotic fetuses. Thus, a larger database would be necessary to better characterize the distributions of both classes. Furthermore, we use 15 DUS templates in our simulations. Although the use of these templates result in variation of the simulated DUS waves, using a larger number of DUS envelopes as templates might produce more realistic DUS simulations.

Secondly, our model only controls the PSD and ApEn of the simulated $FHR_{RRI}$. However, it is important to highlight that the power in each band was controlled independently of the others. The fact that the LF/(MF + HF) ratio have a defined distribution suggests that these features are correlated in a way. Our model did not consider this correlation in the features, which resulted in differences between the target distribution and the obtained distribution as shown in Table 1. This limitation might have an impact on the interpretation of the results that correspond to the LF/(MF + HF) ratio. In addition, there are many other fHRV features that are used to characterize the variability of FHR signals, namely the short-term variability, long term irregularity, the root mean square of successive differences, among others. It is clear that an ideal simulation would be able to account for all the relevant fHRV features and generate as realistic simulations as possible. Nevertheless, we consider that information available in the PSD of the FHR signal is relatable to some of the time-domain features; the LF power carries information about the long-term evolution of the signal, and the HF power carries information about the short-term beat-to-beat variability of the signal. Similarly, nonlinear indicators of signal irregularity can be related to the ApEn of the signal. Thus, although our study does not control nor account for all the features used in the literature, we consider that our results provide a representative understanding of how different fHRV features behave in response to the AC method and the SNR.

Thirdly, our model does not account for signal loss. Implementing signal loss requires to add a different noise model, which behaves as a switch between signal and no signal. We consider that a future study could expand our model to include such a switch using information from real databases. Parameters such as number of drops, or the duration of the artifact can be characterized in their distributions to generate a realistic DUS signal and $FHR_{DUS}$ estimation.

Fourthly, our model does not account for the nonstationary behavior of intrapartum FHR signals. The simulated FHR signals were time invariant within a window of 10 min. However, real FHR signals are nonlinear and time-varying. For a single subject, it is expected that the fHRV distribution will vary across labor: increased uterine activity will generate responses in the FHR and fHRV (Warrick and Hamilton, 2012; Lear et al., 2018). Thus, the length of analysis window is an important parameter to consider and optimize: short analysis windows will provide large variability in the estimated features, while long analysis windows will include nonstationary FHR. Another factor that will affect the estimated fHRV is fetal state. It has been shown that fetuses have variable fHRV distributions when they are in quiet and active periods (Signorini et al., 2003). Thus, a long analysis window might contain more than one fetal state, which is also nonstationary behavior. An alternative approach to optimizing the length of the analysis window is to consider time-varying or parameter-varying models to describe FHR and fHRV. Future studies should consider these models in the analysis of intrapartum FHR.

Finally, our model does not consider the effect of gestational age (GA). It has been reported that the distribution of fHRV features vary with GA (Gonçalves et al., 2018). However, no significant difference was reported in PSD or ApEn features for term infants (GA > 36 weeks). Considering that our simulations took as reference fHRV distributions from term infants or intrapartum signals, we believe that our results are valid regardless GA in term infants. Further studies should analyze if the trends of the bd and rd are different when GA < 36 weeks.

## Conclusion

Our results demonstrate the susceptibility of fHRV features to the AC method and additive noise in the clinical acquisition of FHR. The dependency of the estimation error on the AC window length, which is part of the proprietary information of the FHR monitor manufacturers, is a limitation in comparing data acquired from different monitors. There is an increasing interest in applying machine learning techniques to FHR tracings on large databases to identify fetuses at risk during antepartum (Signorini et al., 2020) and intrapartum monitoring (Georgieva et al., 2017; Petrozziello et al., 2019). Although the discriminability of fHRV features depends on the AC window length of the FHR monitor and the SNR, it has low variability (<5.4%). Moreover, a feature based on the power below 72 mHz is not affected by the AC method. Thus, understanding the effects of the AC method on fHRV discriminability would potentially lead to a better implementation of ML classifiers of FHR signals when dealing with multiple sources. LF power, MF power, and the LF/(MF + HF) ratio are least affected by the AC method in average but are more influenced by changes in the AC window length and SNR. Classifiers based on these features would benefit from including the fetal monitor model, or acquisition center, as part of the regressor. On the other hand, HF power and ApEn experience the largest loss of discriminability in average, but with lower dependency on AC window length and SNR. Thus, classifiers based on these features would not need to account for differences in the acquisition fetal monitors.

## Data Availability

The fetal ECG signals used in this article are openly available as part of the Noninvasive Fetal ECG - The PhysioNet Computing in Cardiology Challenge 2013 (Accession Number: 25401167, URL: https://physionet.org/content/challenge-2013/1.0.0/) and the Abdominal and Direct Fetal ECG Database (Accession Number: 25854665, URL: https://physionet.org/content/adfecgdb/1.0.0/).
